# Evaluation of a Fascial Sling Technique for Primary Modiolus Reconstruction to Preserve Lip Symmetry Following Head and Neck Free Flap Surgery: A Preliminary Report

**DOI:** 10.1002/micr.70308

**Published:** 2026-11-08

**Authors:** Fuko Omine, Kaoru Sasaki, Junya Oshima, Hironao Hanihara, Yukiko Aihara, Yoichiro Shibuya, Mitsuru Sekido

**Affiliations:** ^1^ Department of Plastic, Reconstructive and Hand Surgery University of Tsukuba Tsukuba Ibaraki Japan

## Abstract

**Background:**

Modiolus loss after ablative head and neck surgery can result in oral incompetence, commissure deviation, and poor facial symmetry. Although fascial suspension for perioral reconstruction has been described, the value of incorporating primary modiolus reconstruction into free flap surgery has not been well defined. This report evaluated the feasibility and postoperative outcomes of primary modiolus reconstruction using a fascial sling.

**Methods:**

We retrospectively reviewed 13 patients who underwent unilateral primary modiolus reconstruction during head and neck free flap surgery between June 2010 and August 2024. Fascia harvested from the flap donor site was inset in a Y‐ or V‐shaped configuration to suspend the upper and lower lips from the masseter muscle. Outcomes included commissure deviation at rest and during mouth opening evaluated using a quantitative photographic method, flap‐ and donor‐site complications, commissure ptosis, and diet.

**Results:**

Thirteen patients with a mean age of 72.5 years (range, 61–86 years) underwent reconstruction; 11 had buccal mucosal carcinoma; 1, lower lip carcinoma; and 1, accessory parotid gland carcinoma. All the patients underwent ALT flap reconstruction; the fascial configuration was Y‐shaped in 10 patients and V‐shaped in 3 patients. Eleven patients were evaluable at least 6 months postoperatively. At rest, seven patients showed no commissure deviation and four showed mild deviation. During mouth opening, 10 patients were evaluable: 7 showed no deviation; 2, mild deviation; and 1, moderate deviation; no patient showed severe deviation. No flap‐related or donor‐site complications or postoperative commissure drooping were observed. Four patients resumed a regular diet; five, a soft diet; and four, a pureed diet.

**Conclusion:**

Primary modiolus reconstruction may help preserve oral commissure position and symmetry, with a low risk of procedure‐related complications. Considering survival outcomes, technical advantages, and surgical invasiveness, performing modiolus reconstruction at the time of the initial surgery may be clinically meaningful.

## 1 Introduction

The modiolus is the site where several perioral muscles, including the orbicularis oris and buccinator muscles, converge. In head and neck surgery for tumors such as buccal mucosal carcinoma, the modiolus is often resected. As a result, the functional balance of the lips is lost in the reconstructed lip lacking the modiolus (Sasaki et al. 2020). In other words, the oral commissure deviates toward the unaffected side during movement, resulting in both functional and esthetic problems.

Although the usefulness of modiolus reconstruction has become increasingly recognized in recent years, the number of reports remains limited, and no studies have examined the timing of reconstruction; therefore, no consensus has been reached regarding the optimal timing of reconstruction.

The purpose of this report was to retrospectively evaluate the functional and esthetic outcomes and complications in patients who underwent primary modiolus reconstruction and to clarify its clinical significance and associated challenges.

## 2 Patients and Methods

The cases of 13 patients who underwent unilateral primary modiolus reconstruction concurrently with free tissue transfer for head and neck reconstruction at the University of Tsukuba Hospital between June 2010 and August 2024 were retrospectively reviewed. The follow‐up period ranged from 1 to 96 months postoperatively. The variables investigated included age, sex, primary disease, tumor stage, type of flap, site of fascial harvest, fascial configuration, method of oral commissure reconstruction, postoperative radiotherapy, and prognosis. The outcome measures were oral commissure deviation at rest and during mouth opening, complications of the flap and donor site, the presence or absence of drooping of the oral commissure on the affected side, and diet.

This investigation was conducted with the approval of the ethics committee of the University of Tsukuba Hospital.

## 3 Surgical Technique

Modiolus reconstruction is a procedure in which the stumps of the orbicularis oris muscle in the upper and lower lips are suspended from the masseter muscle in a Y‐shaped or V‐shaped fashion using fascia.

In cases involving an anterolateral thigh (ALT) flap, the fascia used for suspension is harvested concurrently as fascia lata from the flap donor site. When sufficient fascial length cannot be obtained, the fascia is harvested with a greater width and arranged in a Y‐shaped configuration; when sufficient fascial width cannot be obtained, it is harvested with a greater length and arranged in a V‐shaped configuration. In either case, the fascia is adjusted so that its final width is at least 5–10 mm.

Next, the orbicularis oris muscle is sutured to the fascia. This step is often performed after the rough inset of the flap and completion of vascular anastomosis. In the Y‐shaped configuration, one end of the fascia is split into two limbs to create a Y shape; the two divided ends are sutured to the oral commissure, and the opposite unsplit end is anchored to the masseter muscle. In the V‐shaped configuration, the central portion of a single straight fascial strip is passed around the masseter muscle in a hooking fashion, and both ends are sutured to the upper and lower lips, respectively. In either method, the lips are suspended using the masseter muscle as a supporting point. Depending on the flap design, the procedure may also involve passing the fascia through the flap (Figure 1).

**FIGURE 1:**
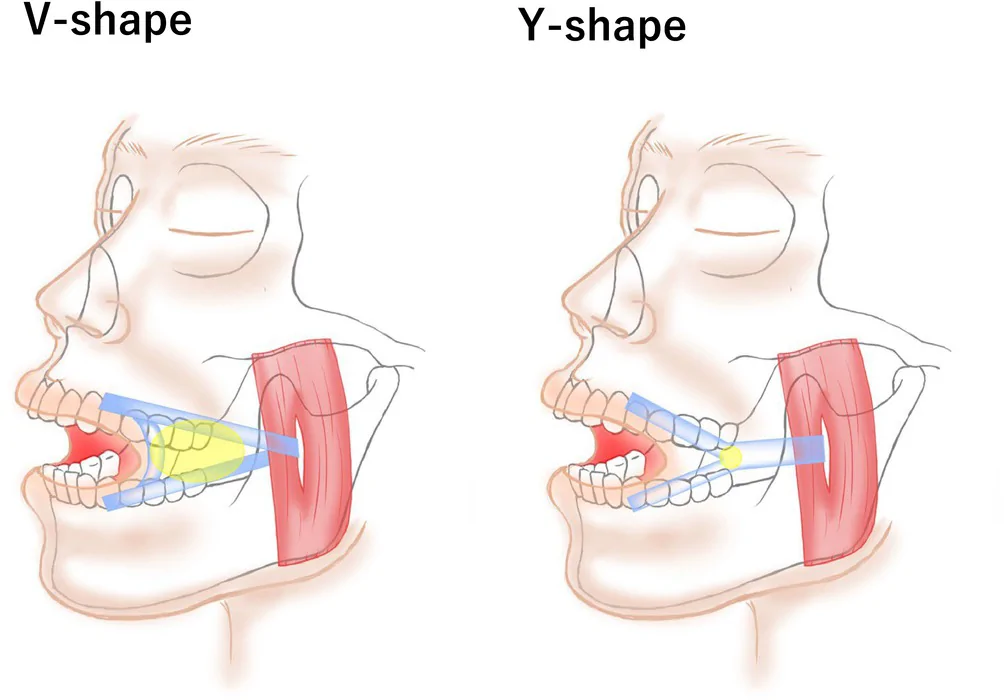
Y‐shaped and V‐shaped fascial sling suspension. The Y‐shaped sling is fixed to the oral commissure and masseter muscle, whereas the V‐shaped sling is looped around the masseter muscle and fixed to the upper and lower lips. Reproduced from Sasaki et al. (2020) with permission from Wiley Periodicals Inc.

## 4 Quantitative Evaluation of Deviation

Because no established method for evaluating oral commissure deviation is available, we defined a quantitative evaluation method for this investigation. Frontal photographs taken during outpatient follow‐up, both with the mouth open and with the mouth closed, were used for the evaluation. A line connecting the bilateral medial canthi was defined as the horizontal reference line, and a perpendicular line passing through its midpoint was defined as the midline. In cases in which the medial canthi were not included in the photographic field, a line connecting the inferior ends of both earlobes was used as a substitute horizontal reference line. The horizontal distance from the midline to each oral commissure was measured, with the affected side designated as *a* and the unaffected side as *b*. The ratio *a*/*b* was defined as the oral commissure deviation ratio; 0.9 ≤ *a*/*b* was classified as no deviation or deviation toward the affected side; 0.7 ≤ *a*/*b* < 0.9, as mild; 0.5 ≤ *a*/*b* < 0.7, as moderate; and *a*/*b* < 0.5, as severe. These evaluations were performed separately with the mouth closed and with the mouth open (Figure 2).

**FIGURE 2:**
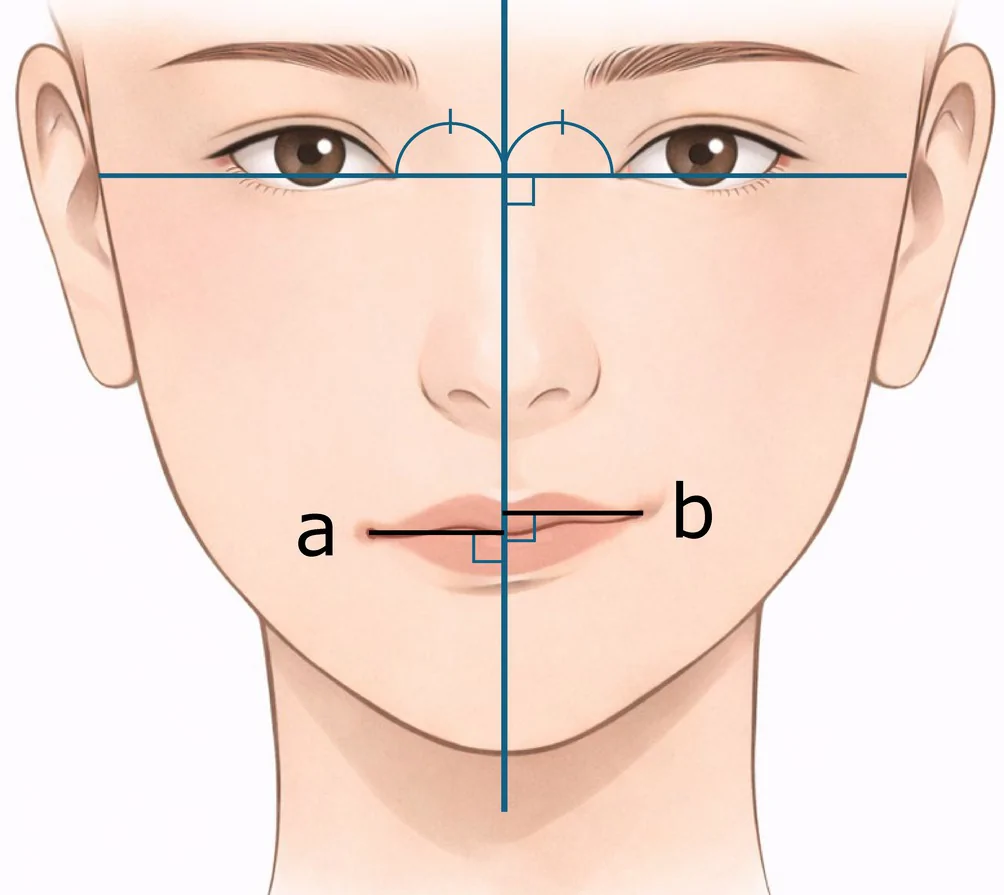
The midline of the face is defined as the line drawn vertically from the midpoint of the line connecting the inner canthi of the eyes. A line is drawn through the shortest distance from the midline to each corner of the mouth, designating the affected side as *a* and the unaffected side as *b*.

## 5 Results

The mean age of the patients was 72.5 years (range, 61–86 years); 5 of the 13 patients were male. The primary diseases were buccal mucosal carcinoma in 11 patients, lower lip carcinoma in 1 patient, and accessory parotid gland carcinoma in 1 patient. The tumor stage was T4 in five patients, T3 in seven patients, and T2 in one patient. In patients with multiple primary cancers, the cases were classified as T3 on the basis of the highest T classification. All the reconstructions were performed using an ALT flap, and the fascia used for modiolus reconstruction was harvested from the fascia lata at the flap donor site in all the cases. Reconstruction of the oral commissure skin was performed using a flap in three patients and direct closure in six patients, and was not required in four patients. The fascial configuration for modiolus reconstruction was V‐shaped in 3 patients and Y‐shaped in 10 patients. Postoperative radiotherapy was administered in six patients. During follow‐up, recurrence of the malignant tumor was observed in six patients. No secondary local or regional flap procedures were performed in any of the patients.

Oral commissure deviation was evaluated in 11 patients who were followed for at least 6 months postoperatively. With the mouth closed, oral commissure deviation was classified as no deviation in seven patients and as mild in four patients. With the mouth open, oral commissure deviation was classified as no deviation in seven patients, as mild in two patients, and as moderate in one patient. One patient was excluded because an appropriate photograph with the mouth open was not available. No patient was classified as having severe oral commissure deviation with either the mouth closed or the mouth open. No flap‐related or donor‐site complications were observed in any of the 13 patients, and no patient developed drooping of the oral commissure on the affected side. Regarding diet, four patients resumed a regular diet; five, a soft diet; and four, a pureed diet. Patient‐level clinical characteristics, treatment details, quantitative photographic evaluation data, and functional outcomes are summarized in Table 1.

**TABLE 1:** Clinical characteristics, treatment details, quantitative photographic evaluation of the oral commissure position, and functional outcomes.

Case	Age	Sex	Primary diagnosis	Stage	Flap	Fascia placement	Lip reconstruction	RT	Oral commissure deviation				Flap and donor site complications	Drooping	Diet	Follow‐up (months)	Outcome
									Closed	CDR	Open	CDR					
1	70	F	Buccal mucosa carcinoma	T3	ALT	V	Flap	Yes	None	1.15	None	1.10	None	None	Regular	7	Transferred
2	65	F	Lower lip carcinoma	T3	ALT	V	Flap	No	None	0.95	None	1.04	None	None	Regular	19	
3	73	F	Buccal mucosa carcinoma	T4a	ALT	Y	Primary closure	No	Mild	0.76	Moderate	0.65	None	None	Regular	96	Follow‐up ended
4	86	F	Buccal mucosa carcinoma	T4a	ALT	Y	Primary closure	Yes	NE		NE		None	None	Soft	1	Recurrence/death
5	72	F	Buccal mucosa carcinoma	T4b	ALT	Y	Primary closure	Yes	Mild	0.82	None	0.93	None	None	Soft	10	Recurrence/death
6	77	F	Buccal mucosa carcinoma	T3	ALT	Y	Flap	No	None	0.95	None	0.93	None	None	Pureed	33	
7	71	M	Buccal mucosa carcinoma	T4a	ALT	Y	Primary closure	No	Mild	0.84	None	0.90	None	None	Regular	36	
8	78	F	Buccal mucosa carcinoma	T2	ALT	V	Primary closure	No	Mild	0.87	Mild	0.78	None	None	Soft	48	
9	73	M	Buccal mucosa carcinoma	T3	ALT	Y	None	No	None	1.08	None	0.92	None	None	Soft	36	
10	61	M	Buccal mucosa carcinoma	T3	ALT	Y	None	Yes	None	1.27	Mild	0.70	None	None	Pureed	6	Recurrence/death
11	72	F	Carcinoma of floor of mouth/buccal mucosa/tongue	T3	ALT	Y	None	Yes	None	0.91	None	1.00	None	None	Pureed	6	Recurrence
12	65	M	Buccal mucosa carcinoma	T3	ALT	Y	Primary closure	No	NE		NE		None	None	Pureed	1	Recurrence/death
13	79	M	Accessory parotid gland carcinoma	T4a	ALT	Y	None	Yes	None	1.16	NE		None	None	Soft	16	Recurrence/death

*Note:* CDR was defined as the ratio of the perpendicular distance from the facial midline to the affected oral commissure (*a*) to the corresponding distance on the contralateral side (*b*), calculated as *a*/*b*. Mouth opening in patient No. 13 could not be evaluated because no open‐mouth photograph was available. Other missing values were excluded from evaluation because the patients died within 6 months of surgery.

Abbreviations: ALT, anterolateral thigh flap; CDR, commissure deviation ratio; NE, not evaluated; RT, radiotherapy.

## 6 Case Report

A 71‐year‐old man underwent reconstruction of a full‐thickness buccal defect involving the oral commissure following resection of buccal mucosal carcinoma (T4aN0M0) (Figure 3A). An ALT flap was harvested for reconstruction (Figure 3B), and fascia lata was concurrently harvested for suspension (Figure 3C). After the flap was inset into the skin defect and vascular anastomosis was completed, modiolus reconstruction was performed (Figure 3D,E). The fascia was passed through the flap and arranged in a Y‐shaped configuration to suspend the oral commissure. At 3 years postoperatively, oral commissure deviation was assessed as mild with the mouth closed (*a*/*b* = 0.84) and as absent with the mouth open (*a*/*b* = 0.90) (Figure 3F,G).

**FIGURE 3:**
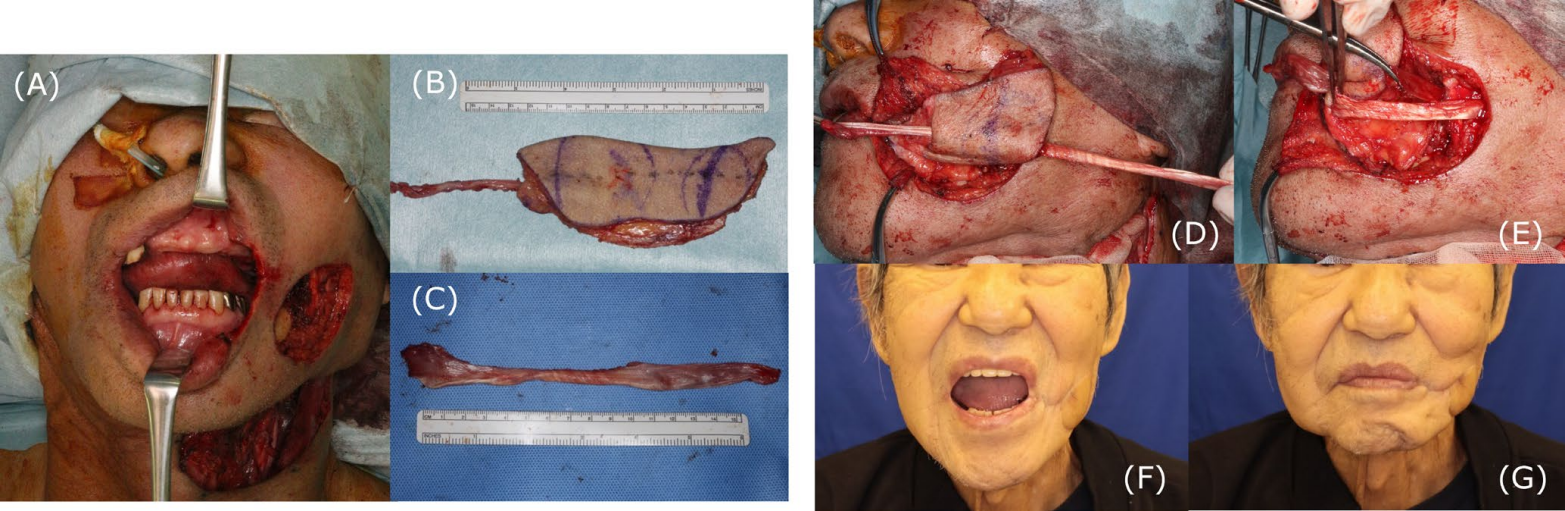
Surgical procedure and postoperative outcomes. (A) Intraoperative view after tumor resection. (B) Harvested anterolateral thigh (ALT) flap. (C) Harvested fascia lata for fascial sling. (D) After preliminary inset of the flap, a subcutaneous tunnel was created within the flap by blunt dissection, and the fascial sling was passed through the tunnel. (E) After the orbicularis oris muscle was sutured to the fascial sling, the sling was passed around the masseter muscle, and the appropriate traction tension was adjusted. (F) Postoperative appearance during mouth opening. (G) Postoperative appearance at rest.

## 7 Discussion

Fascial suspension for the lips has long been performed. Conventionally, static suspension methods using the palmaris longus tendon or fascia lata have mainly been employed (Arem 1975; Serletti et al. 1997; Jeng et al. 2004; Kuo et al. 2008; Huang et al. 2016). In Maegawa et al. (1999) reported a transverse fascial sling for facial palsy. Thereafter, Sasaki et al. (2012) applied this method to head and neck reconstruction and reported it as modiolus reconstruction. This method, in which the upper and lower lips are suspended laterally from the masseter muscle, cannot be regarded as completely dynamic reconstruction; nevertheless, because it moves to some extent in coordination with the movement of the masseter muscle, it may be considered more dynamic than other static suspension methods.

In the present technique, the residual orbicularis oris muscles of the upper and lower lips are connected to a fascial sling at the reconstructed oral commissure, and the sling is anchored to the masseter muscle. This configuration reconstructs key functions of the modiolus, including force convergence at the oral commissure, lateral support, and coordinated tension between the upper and lower lips. Thus, the term “modiolus reconstruction” in this report refers to functional reconstruction of the modiolar mechanism.

Regarding the choice of fascia, we used a separately harvested nonvascularized fascia lata graft rather than incorporating vascularized fascia into the ALT flap. This approach provides greater flexibility in determining the course, fixation points, and tension of the sling and avoids transmitting traction or compression to the vascular pedicle. Furthermore, even when the fascia remains continuous with the flap, reliable perfusion throughout a long, narrow fascial strip configured as a sling cannot necessarily be assumed, particularly because the sling is placed under sustained tension. For these reasons, we elected to harvest the fascia lata separately from the free flap.

Previous reports have described both primary and secondary modiolus reconstruction. However, to our knowledge, no previous study has specifically examined the significance of performing modiolus reconstruction primarily at the time of free‐flap reconstruction. The novelty of the present report lies in two aspects: first, it evaluates primary modiolus reconstruction as part of the initial free‐flap procedure; second, it quantitatively assesses postoperative oral commissure position using photographic measurements rather than relying solely on subjective evaluation.

In this series of 13 patients, symmetry at rest was generally well preserved, and no patient showed severe deviation. During mouth opening, deviation tended to be greater than at rest. In addition, patients in whom the oral commissure was reconstructed by direct closure tended to show deviation toward the unaffected side. However, because of the limited number of patients, these findings should be interpreted cautiously.

Furthermore, no flap‐related or donor‐site complications were observed with this technique, suggesting that it can be performed safely. In addition, because drooping of the oral commissure on the affected side was not observed, the technique was considered to provide effects comparable to those reported previously for modiolus reconstruction.

From a technical perspective as well, performing modiolus reconstruction primarily offers substantial advantages. At the time of the initial operation, the anatomic structures are easier to identify within normal tissue, and tissue flexibility is still preserved. In addition, after tumor resection, the surgical field is widely exposed, allowing the route of fascial placement to be adjusted largely under direct vision.

Furthermore, regarding the donor site, the procedure can be accomplished simply by additionally harvesting 15–20 cm of fascia or tendon tissue from the same operative field at the time of flap harvest. In contrast, secondary modiolus reconstruction requires a new donor site.

It is widely recognized that surgery in scarred tissue is more difficult than surgery in normal tissue. Moreover, in secondary modiolus reconstruction, the route for fascial placement often has to be created blindly. Therefore, compared with primary modiolus reconstruction, which can be performed while confirming the structures under direct vision, secondary modiolus reconstruction is considered to be technically more demanding and to carry a higher risk of vascular injury.

Many patients undergoing head and neck reconstruction are likely to receive postoperative radiotherapy. One report indicated that approximately 78.6% of patients with oral cancer classified as T1–2N1 (corresponding to Stage III) underwent radiotherapy (Shrime et al. 2010). In our series, 6 of 13 patients received postoperative radiotherapy. Tissue after irradiation is likely to become stiffer and more fragile than ordinary scar tissue, which may represent another important reason to perform modiolus reconstruction during the initial reconstructive surgery.

Regarding operative time, fascia harvest can be performed within the same operative field used for flap elevation, and the suspension procedure itself can generally be completed with approximately 15 min of additional planning and operative manipulation during flap inset. Therefore, incorporating this technique as part of the initial reconstruction is not considered particularly difficult.

From the perspective of survival outcomes, advanced buccal mucosal carcinoma, which accounted for the majority of cases in the present series, has been reported to have a 5‐year survival rate of approximately 56% for Stage III disease and 43% for Stage IV disease (Bobdey et al. 2018). Furthermore, in the report by Dekerle et al. (2021), among patients who underwent free flap reconstruction following head and neck cancer resection, the subgroup identified by multivariate analysis as having poor prognostic factors (T3–4 disease and age ≥ 60 years) was estimated, on the basis of the presented survival curves, to have a 5‐year survival rate of approximately 25%, indicating an extremely poor prognosis.

In addition, most recurrences have been reported to occur within 1 year of surgery (Struckmeier et al. 2024). In our series, recurrence occurred in 6 of the 13 patients, all within 1 year of surgery.

Considering these survival outcomes and the timing of recurrence, a substantial proportion of patients may not have sufficient time to wait for secondary reconstruction. Therefore, in cases likely to require modiolus reconstruction, it may be meaningful to consider primary modiolus reconstruction at the time of the initial surgery rather than assuming secondary reconstruction.

In the present investigation, no complications related to this technique, such as flap circulatory compromise, were observed. In addition, although the surgical details differed, previous reports describing primary modiolus reconstruction using an ALT flap combined with vascularized fascia or a radial forearm flap combined with the palmaris longus tendon noted complications including venous congestion, cervical hematoma formation, and wound infection (Jeng et al. 2004; Kuo et al. 2008). However, flap survival was 100% in all these reports. Taken together, these findings suggest that no major complications occurred in the present series and that this technique is clinically feasible.

Therefore, in cases involving modiolus defects, primary modiolus reconstruction is considered to be an option worthy of consideration.

The photographic evaluation in the present report was performed retrospectively, and the photographs had not been obtained in a standardized manner specifically for evaluation purposes. Therefore, some variability in the photographic assessments was unavoidable.

In addition, this report was a single‐arm case series, and comparison with cases without modiolus reconstruction would be desirable. Furthermore, the number of cases was not sufficiently large. Future investigations will require a larger number of cases, as well as evaluation of long‐term stability and dynamic functions such as speech and mastication.

Moreover, in pursuit of more natural lip function, reconstruction of depressor muscle function, which cannot be adequately reproduced by lateral suspension alone, should also be investigated in the future.

## 8 Conclusion

Primary modiolus reconstruction may help preserve oral commissure position and symmetry, with a low risk of procedure‐related complications. Considering survival outcomes, technical advantages, and surgical invasiveness, performing modiolus reconstruction at the time of the initial surgery may be clinically meaningful.

## Author Contributions

**Fuko Omine:** methodology, investigation, data curation, visualization, writing – original draft, writing – review and editing. **Kaoru Sasaki:** conceptualization, investigation, supervision, writing – review and editing. **Junya Oshima:** investigation. **Hironao Hanihara:** investigation. **Yukiko Aihara:** investigation. **Yoichiro Shibuya:** investigation. **Mitsuru Sekido:** investigation. All authors critically reviewed and approved the final version of the manuscript and agreed to be accountable for all aspects of the work.

## Funding

The authors have nothing to report.

## Ethics Statement

This retrospective observational study was approved by the Institutional Review Board of Ethical Committee, University of Tsukuba Hospital (approval number: R06‐255). The requirement for individual written informed consent was waived by the Institutional Review Board because of the retrospective nature of the study. Information about the study was made available on the institutional website, and patients were provided with the opportunity to opt out of the study.

## Conflicts of Interest

The authors declare no conflicts of interest.

## Data Availability

The data that support the findings of this study are available on request from the corresponding author. The data are not publicly available due to privacy or ethical restrictions.
